# Probing the Feasibility of Single-Cell Fixed RNA Sequencing from FFPE Tissue

**DOI:** 10.3390/ijms27031605

**Published:** 2026-02-06

**Authors:** Xiaochen Liu, Katherine Naughton, Samuel D. Karsen, Patricia Bentley, Lori Duggan, Neha Chaudhary, Kathleen M. Smith, Lucy Phillips, Dan Chang, Naim A. Mahi

**Affiliations:** 1 AbbVie, Cambridge Research Center, 200 Sidney Street, Cambridge, MA 02139, USA; 2 AbbVie Bioresearch Center, 100 Research Drive, Worcester, MA 01605, USA

**Keywords:** single-cell RNA-seq, FFPE, FFPE-seq

## Abstract

Single-cell RNA sequencing (scRNA-seq) provides a comprehensive understanding of cellular complexity; however, its requirement for fresh or frozen samples limits its flexibility. To overcome this limitation to effectively leverage clinical samples, Chromium Fixed RNA Profiling on formalin-fixed paraffin-embedded (FFPE) tissue blocks (scFFPE-seq) was developed to perform single-nucleus RNA sequencing from nuclei isolated from FFPE. In this study, we utilized fresh tissue samples from colon, ileum, and skin to assess the viability of scFFPE-seq compared to these fresh samples. We were able to recover unique cell types from challenging FFPE tissues and validated scFFPE-seq findings through Hematoxylin and Eosin (H&E) images. The results demonstrated that scFFPE-seq effectively captured the single-cell transcriptome in FFPE tissues, obtaining comparable cell abundance, cell type annotation, and pathway characterization to those in fresh tissues. Overall, the study presents strong evidence of the potential of scFFPE-seq to enhance scientific knowledge by enabling the generation of high-quality, sensitive single-nucleus RNA-seq data from preserved tissue samples. This technique unlocks the vast archives of FFPE samples for extensive retrospective genomic studies.

## 1. Introduction

Single-cell RNA sequencing (scRNA-seq) is a cutting-edge technology that has emerged in recent years, offering a significant advantage compared to bulk RNA-seq by analyzing transcriptomics at a finer resolution, which allows for a more comprehensive understanding of cellular complexity and heterogeneity [1,2,3]. However, scRNA-seq is limited by the need for freshly collected samples and immediate processing [4,5,6,7]. Such time-sensitive requirements can be challenging and restrict the flexibility of study design, especially for tissue samples that are difficult to collect. To overcome such challenges, new technologies have been developed to perform single-nucleus RNA sequencing from nuclei isolated from formalin-fixed paraffin-embedded (FFPE) tissue blocks, including scFFPE-seq [5,8], snFFPE-seq [9], snRandom-seq [10], and snPATHO-seq [11].

scFFPE-seq uses Chromium Single-Cell Gene Expression Flex, which utilizes probe hybridization rather than poly-A capture to profile the whole transcriptome with improved sensitivity over the 3′ end. This method offers a highly sensitive and efficient approach to extract and sequence nuclei from FFPE samples, expanding opportunities to investigate archival collections, as well as facilitating use in longitudinal cohorts [12,13,14,15]. Another advantage of this method is that it captures cells, such as neutrophils and adipocytes, which are difficult to preserve in fresh tissue but can be detectable from fixed tissue. Despite its potential, limited evaluation has been reported in the literature. Trinks et al. [6] benchmarked gene expression profiles from fresh, cryopreserved, and FFPE lung cancer tissues, and suggested scRNA-seq from FFPE tissues can serve as a substitute for fresh tissue, with comparable results.

In this study, we examined the feasibility of using scFFPE-seq on various human FFPE tissues, including colon, ileum, and skin samples. The results demonstrate the versatility and reliability of scFFPE-seq across a range of tissue types. Additionally, our study demonstrated the capability of scFFPE-seq to recover unique cell types from FFPE tissues, such as neutrophils and adipocytes. The validation of scFFPE-seq findings through H&E images and its comparability to fresh scRNA data further emphasizes its effectiveness in capturing the cellular biology within the tissue microenvironment. Compared to total-RNA profiling methods like snPATHO-seq [11] or snRandom-seq [10]—which may provide broader transcript recovery—scFFPE-seq offers a streamlined, scalable workflow that is compatible with established Chromium Flex technologies and is well-suited for applications where cellular resolution and validated marker detection are prioritized over absolute transcriptome breadth. Similarly to snPATHO-seq or snRandom-seq, scFFPE-seq achieves good concordance in cell abundance and transcriptional signatures compared to fresh or frozen references.

Overall, our study provides strong evidence that this technology shows great potential in advancing scientific knowledge and facilitating generation of high-quality and sensitive single-nucleus RNA-seq from a variety of preserved tissue samples, including commercial archival tissues. It unlocks the vast archives of FFPE tissues, allowing for extensive retrospective clinical genomic studies. This opens new opportunities to explore the molecular profiles of archived samples and their correlation with clinical outcomes [4,6,7], providing valuable insights for research and potentially transforming personalized medicine approaches.

## 2. Results

### 2.1. Single-Cell Quality Matrices in FFPE Samples

**Figure 1:**
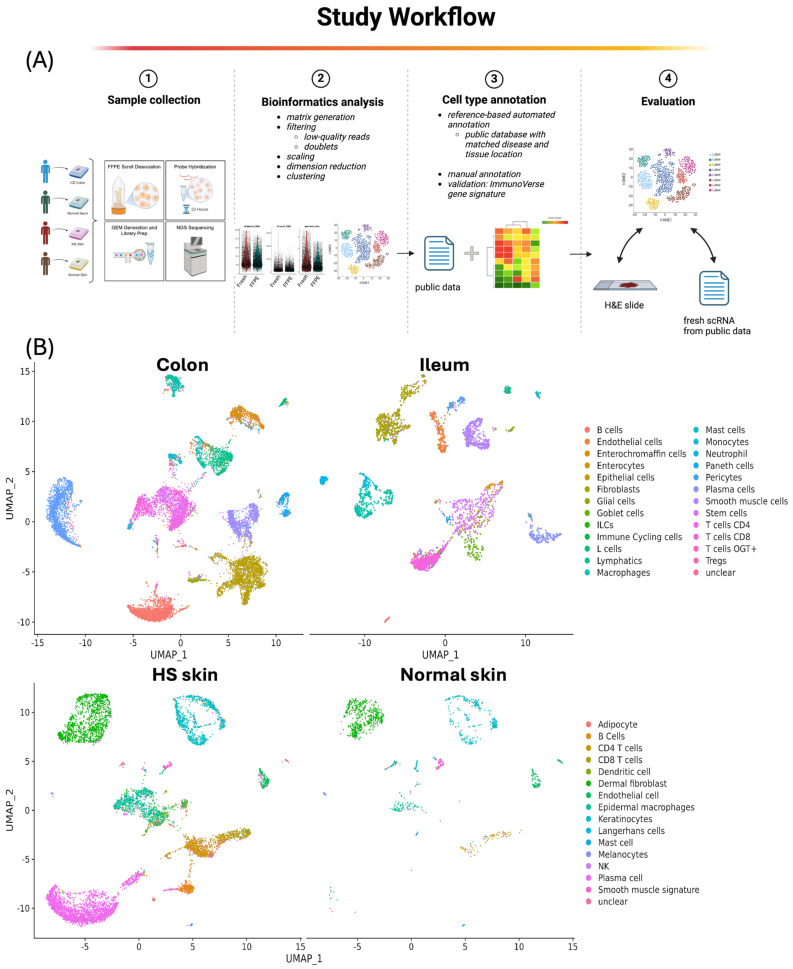
(**A**) Study workflow for cell type annotation and evaluation. The process begins with FFPE tissue blocks from CD colon, normal ileum, HS skin, and normal skin tissues. Bioinformatics analysis follows, involving matrix generation, filtering of low-quality reads and doublets, scaling, dimension reduction, and clustering. Subsequently, cell type annotation is conducted through both reference-based automated annotation using public databases tailored to matched disease and tissue location, and manual annotation. Validation is performed using the IBDTransDB gene signature [16]. The evaluation step uses data from H&E slides and fresh single-cell RNA sequencing data obtained from public sources to assess the accuracy and relevancy of the annotated cell types. (**B**) UMAP of major cell types for CD colon, normal ileum, HS skin, and normal skin tissues, colored by cell type. There are 25 major cell types that were identified for colon and ileum, and 14 and 16 cell types from normal and HS skin, respectively.

A total of 31,331 cells were sequenced with an average of 16,519 ± 536 detected genes, using two scrolls for colon and ileum samples and six scrolls for skin samples (Table 1, Figure 1A and Supplementary Figures S1–S5). A total of 26,249 cells from four samples passed the quality control steps, with an average of 84% passing rate. In terms of cell count, the colon sample exhibits the highest cell count with 13,778 cells, followed by HS skin and ileum containing 7705 cells and 3720 cells, respectively.

In contrast, normal skin tissue has the lowest cell count, consisting of only 1406 cells. However, it exhibits the highest sequencing depth with a median of 25,061 reads per cell. The ileum follows with roughly 12,000 reads per cell, while the colon and HS have similar sequencing depths of approximately 6000 to 7000 reads per cell. All four samples have a sequencing saturation with a range of 73–93%. Following use of the analysis pipeline, we identified 25 major cell types for colon and ileum, as well as 14 and 16 cell types from normal and HS skin, respectively (Figure 1B, Supplementary Table S1). Among them, 54 and 106 neutrophil cells were uniquely identified in colon and ileum, respectively. Additionally, 12 adipocyte cells were uniquely identified in HS skin.

### 2.2. Cell Abundance Evaluation in scFFPE-Seq via H&E Imaging

**Figure 2:**
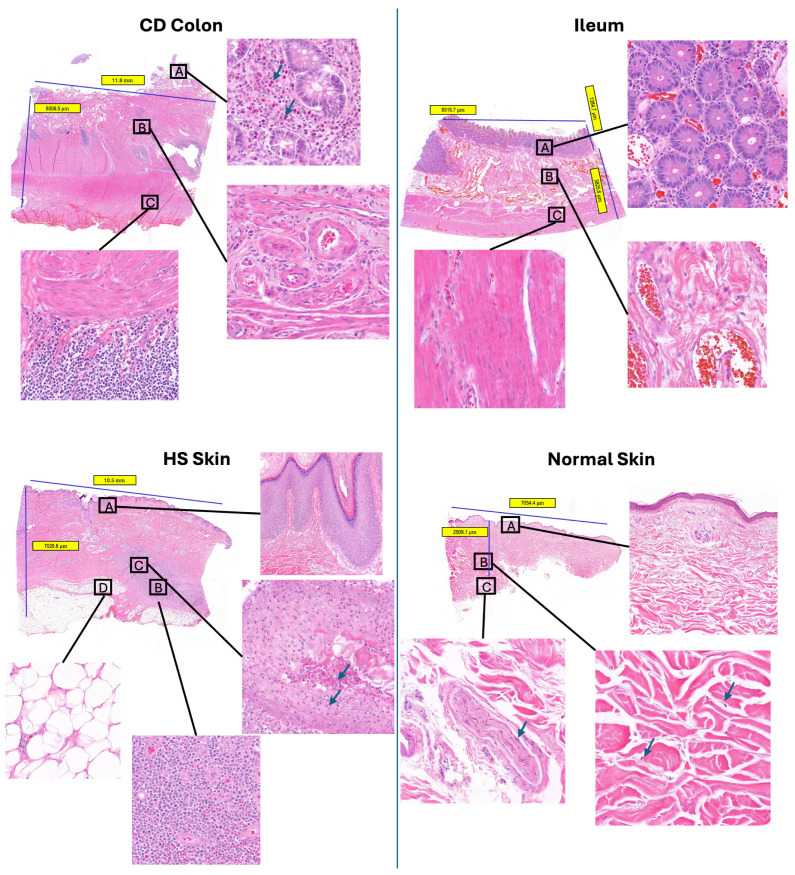
H&E images of Crohn’s disease colon (**upper left**), normal ileum (**upper right**), Hidradenitis Suppurativa skin (**lower left**), and normal skin (**lower right**). **CD colon**: A: The mucosa is mostly missing, but the remnant area contains enterocytes and goblet cells within a lamina propria that is heavily infiltrated by lymphocytes, plasma cells, and eosinophils (arrows). B: Submucosa contains abundant vessels composed of smooth muscle and endothelial cells, separated by fibroblasts, collagen, and immune infiltrates. C: Muscularis externa is composed of smooth muscle cells and infiltrated by abundant immune cells, predominantly lymphocytes and plasma cells. **Normal ileum**: A: The mucosa is composed of epithelial glands within a lamina propria containing small caliber vessels and mild immune infiltrates. B: Submucosa contains abundant vessels composed of smooth muscle and endothelial cells, separated by fibroblasts, collagen, and immune infiltrates. C: Muscularis externa is composed of smooth muscle cells. **HS skin**: A: Epidermis is hyperplastic with prominent Rete Ridges. B: The deep dermis contains a mixed infiltrate of immune cells with numerous plasma cells. C: Epithelial tendrils composed of keratinocytes extend into the deep dermis and show transmigration of neutrophils into the lumen (arrows). D: The hypodermis contains abundant adipocytes. **Normal skin**: A: Normal epidermis (composed of keratinocytes) and superficial dermis (composed of fibroblasts, collagen, and small caliber blood vessels). B: The deep dermis of normal skin contains abundant collagen with low numbers of fibroblasts (arrows). C: Peripheral nerves (containing Schwann cells, arrows) and blood vessels are also present in the deep dermis.

To obtain deeper insights into cellular composition, we compared cell abundance derived from FFPE samples with those observed in their corresponding H&E images (Figure 2). The results obtained from scFFPE-seq showed strong alignment with the morphological observations from H&E staining. In the CD colon sample, much of the mucosa was absent, with the remaining areas populated with enterocytes and goblet cells, heavily infiltrated by lymphocytes, plasma cells, and eosinophils. The submucosa contained numerous vessels consists of smooth muscle and endothelial cells, along with fibroblasts, collagen, and immune cells. The muscularis externa was dominated by smooth muscle cells, infiltrated by lymphocytes and plasma cells. These findings align with scFFPE-seq data, which confirmed the presence of most cell types and highlighted the low number of epithelial cells alongside a high density of smooth muscle cells. Likewise, in the ileum, the epithelial glands were enveloped by endothelial cells and plasma cells, while the submucosal layer presented a complex matrix containing endothelial cells, fibroblasts, smooth muscle cells, and neutrophils, that were all successfully identified using scFFPE-seq. The deep dermis in HS skin sample displayed a mixed infiltrate of immune cells, particularly plasma cells, and epithelial tendrils composed of keratinocytes extended into the deep dermis, showing neutrophils’ transmigration into the lumen. The hypodermis was replete with adipocytes. Meanwhile, normal skin samples exhibited typical features with keratinocyte-rich epidermis and a superficial dermis composed of fibroblasts. All of these cell types were also detected in the scFFPE-seq data.

In addition, we were able to conduct an estimation of epithelial cell and neutrophil proportions from intestinal tissue, and the keratinocyte proportion from skin tissues using H&E images (Table 2). The results showed an outstanding consistency between scFFPE-seq and H&E estimates. Specifically, FFPE samples only recovered 16.4% of epithelial cells in the ileum and a mere 0.2% in the colon, and H&E estimation had a similar proportion of 17.2% in the ileum and 0.9% in the colon. For neutrophils, both methods indicated a presence of 0.2% neutrophils in the colon, while in the ileum, scFFPE-seq yielded a slightly higher proportion of 0.7% compared to the H&E estimate of 0.2%. In HS skin, scFFPE-seq identified a keratinocytes proportion of 10.8% and similar estimation of 9.3% using H&E images. However, a difference was observed in normal skin, with H&E estimation indicating a proportion of 16.1%, whereas scFFPE-seq data revealed a higher proportion of 28.1%. Overall, these findings validate the robustness and reliability of scFFPE-seq for studying cell populations across a variety of tissue samples.

### 2.3. Gene Expression Comparison Between scFFPE-Seq and Fresh scRNA

**Figure 3:**
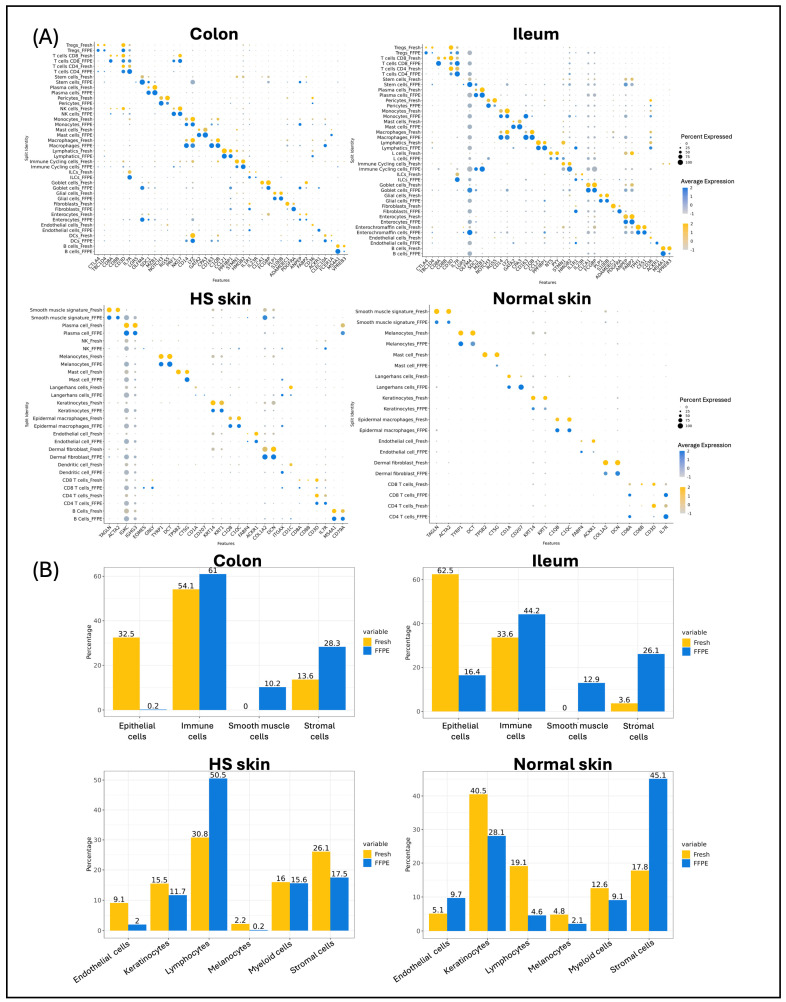
(**A**) Marker gene expression per cell type in fresh (yellow) or FFPE (blue) in each of the tissue types. The darkness or lightness of the color indicates the average expression levels of the genes in each cell type, with darker shades representing higher expression levels. The size of the dots reflects the percentage of cells expressing the marker genes, with larger dots indicating a higher percentage. (**B**) Bar plot of major cell type cellular composition for fresh (yellow) or FFPE (blue) in each of the tissue types. The x-axis categorizes the cell types: epithelial cells, immune cells, smooth muscle cells, and stromal cells, while the y-axis denotes the percentage of each cell type.

We then integrated scFFPE-seq data with public scRNA data from fresh tissue for evaluation. Cell marker genes were similarly expressed between fresh and FFPE across all cell types for all four tissue samples (Figure 3A). In the colon and ileum, we observed trends of marker genes expressing broader and higher in FFPE than fresh data across different cell types, including IL7R, OLFM4, SDC1, CD14, and CD163. This observation may indicate that normal cells are hard to obtain during the dissociation of fresh tissue. In contrast, certain marker genes, such as CD8B for CD8 T cells, were hardly detectable in FFPE samples compared to fresh samples. Other marker genes associated with cell types exhibited high expression levels, validating the accuracy and reliability of the cell type annotation in scFFPE-seq.

**Figure 4:**
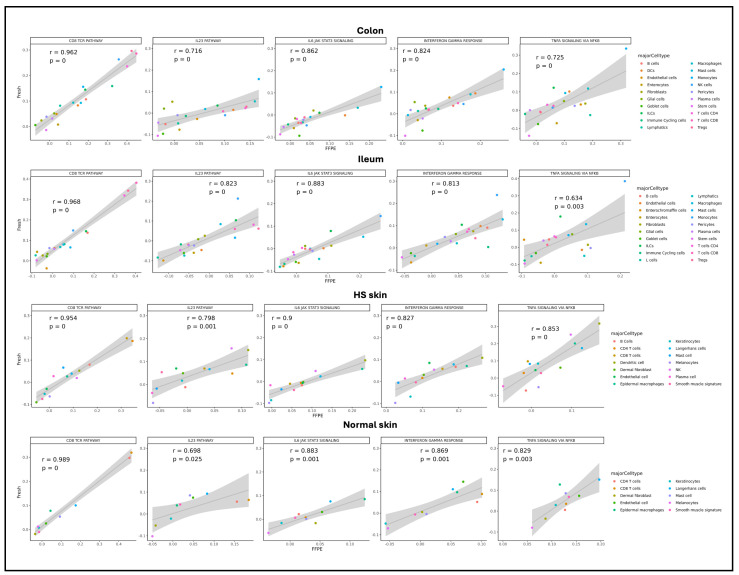
Correlation plot of gene signature scores on biologically relevant selective pathways between fresh (*y*-axis) and FFPE (*x*-axis) samples. Pearson correlation coefficient and *p*-value indicated per gene signature.

We further investigated whether scFFPE-seq enables cell type characterization to a similar extent as fresh scRNA. Gene signature analysis on selective pathways was performed (Figure 4). We found a significantly high correlation between fresh and scFFPE-seq across pathways and tissue types, with Pearson correlations mostly larger than 0.8 (*p*-values ≤ 0.001). Pathway activities were also characterized by cell types, with the CD8/TCR pathway having higher activity in CD8 T cells and CD4 T cells across tissue types. In the colon and ileum, monocytes and macrophages obtained much higher signature scores in related inflammation pathways, such as IL6 JAK/STAT3 signaling, TNF-alpha via NFκB, and interferon-γ response; while in skin it was dendritic cells and epidermal macrophages that showed high activity in the above mentioned pathways (Figure 4).

### 2.4. Cell Abundance in scFFPE-Seq Versus Fresh scRNA

To better compare fresh and FFPE samples, we further grouped the 25 cell types from the colon and ileum into four major cell groups: epithelial cells (enterocytes, goblet cells, and stem cells), immune cells (B cells, dendritic cells (DC), innate lymphoid Cells, immune cycling cells, macrophages, mast cells, monocytes, NK cells, neutrophils, and T cells), stromal cells (endothelial cells, fibroblasts, glial cells, lymphatics, and pericytes), and smooth muscle cells. The results revealed that immune cells showed major similarity between fresh and FFPE samples for both the colon (fresh = 54.1%, FFPE = 61%) and ileum (fresh = 33.6%, FFPE = 44.2%) (Figure 3B, Supplementary Tables S2–S5). We also observed that, stromal cells were strongly enriched in FFPE (28.3% in colon and 26% in ileum) compared to fresh scRNA (13.6% in colon and 3.6% in ileum). In contrast, we found a depletion of epithelial cells in FFPE samples, which is consistent with the results from H&E images.

Next, the 16 cell types identified in skin tissue were grouped into six major cell groups: lymphocytes (T cells, plasma cells, B cells), myeloid cells (langerhans cells, epidermal macrophages, DC, mast cells), stromal cells (dermal fibroblasts, smooth muscle cells, adipocytes), melanocytes, endothelial cells, and keratinocytes. The comparison of FFPE samples from HS skin showed comparable proportions of both keratinocytes and myeloid cells (Figure 3B). In normal skin, only myeloid cells showed similar proportions between the two methods (fresh = 12.6%, FFPE = 9.1%). Melanocytes remained low in abundance with both methods, accounting for less than 5% in both HS and normal skin, with FFPE slightly lower than fresh samples. In contrast, disparities between FFPE and fresh RNA samples were noted in certain cell types. Firstly, the most significant difference in HS skin was observed in lymphocytes, which accounted for 50% in FFPE, much higher than the 38% observed in fresh samples. This difference was primarily driven by the high abundance of plasma cells (Supplementary Table S4). Stromal cells also exhibited differences, with FFPE samples showing a lower proportion of 17.5% compared to 26.1% in fresh scRNA. Upon closer investigation, dermal fibroblasts had relatively similar proportions between FFPE and fresh scRNA (fresh = 18.6%, FFPE = 16.7%), while smooth muscle cells were rarely recovered in scFFPE-seq (0.6%) compared to 7.5% in fresh scRNA. Additionally, endothelial cells were depleted in FFPE (fresh = 9.1%, FFPE = 2%). In addition to HS skin, normal skin also showed some differences in certain cell types. Stromal cells had a strong enrichment of 45% in FFPE compared to 17.8% in fresh scRNA. The proportion of keratinocytes in FFPE samples was found to be 28.1%, which is lower than 40.5% in fresh scRNA data. This is particularly interesting considering that keratinocytes exhibited an overabundance when comparing H&E estimation. Additionally, lymphocytes displayed a lower proportion in FFPE samples (4.6%) compared to fresh scRNA data (19%).

### 2.5. Clinical Conditions in scFFPE vs. Fresh scRNA

We further investigated whether clinical conditions can be characterized at the gene signature level to the same extent in both fresh and FFPE tissue samples. We firstly compared cell abundance between HS and normal skin and found that plasma cells and epidermal macrophages were greatly increased compared to normal skin (Supplementary Figure S6), whereas in normal skin, there was no plasma cell nor B cells detected. Interestingly, dermal fibroblast and keratinocytes were lower in HS skin than normal skin. In addition, we compared gene expression between HS and normal skin. Previous studies using fresh scRNA data reported that, compared to normal skin, HS skin had significantly up-regulated pathways, including tRNA charging, interferon alpha/beta signaling, and interferon gamma signaling. Using same genes associated with these pathways, we found consistent results (Supplementary Figure S7). Genes associated with interferon signaling in HS were higher than normal skin.

## 3. Discussion

scRNA-seq has revolutionized transcriptomic analysis by enabling a finer resolution of cellular complexity and heterogeneity. However, its reliance on freshly collected samples and the need for immediate processing pose limitations, especially when dealing with tissue samples that are difficult to collect. To overcome these challenges, scFFPE-seq was developed, allowing for single-nucleus RNA sequencing of nuclei isolated from FFPE tissue blocks. In this pilot study, we evaluated scFFPE-seq on challenging FFPE tissues including those from the colon, ileum, and skin. Our findings demonstrate that scFFPE-seq can successfully recover unique cell types, with validation corroborated by H&E images. Furthermore, for commonly detected cell types, scFFPE-seq is comparable to fresh scRNA data. These findings highlight the potential of scFFPE-seq to expand research utilizing archival tissue collections and facilitate studies involving longitudinal cohorts.

FFPE sections are commonly used for pathology reviews due to their ability to provide high-quality histology and immunohistochemistry information. This allows for confident regional annotation of FFPE blocks, unlike fresh tissue samples obtained through punch biopsy [17,18,19,20]. In this study, we found that scFFPE-seq via incisional biopsy can successfully recover distinct cell types, such as neutrophils and adipocytes, which are often difficult to capture in fresh scRNA due to their fragility [21]. FFPE dissociation yields a suspension that mostly consists of nuclei with some intact cells. Since the dissociated FFPE suspension mostly contains nuclei, this may allow greater recovery of certain cell types that might not be as effectively captured in a fresh sample, as seen in tissues like neutrophils and adipose. Additionally, smooth muscle cells were better captured by incisional biopsy compared to punch biopsy. H&E images supported scFFPE-seq data, further validating our findings, including epithelial cell and neutrophils from the colon and ileum, as well as neutrophils from HS skin. These cell annotations from scFFPE-seq closely aligned with estimations from H&E analysis. Lastly, we observed an enrichment of stromal cells scFFPE-seq, consistent with previous studies [6]. This suggests that the tissue dissociation stress during fresh RNA sample preparation could be reduced with FFPE, which appears effective at preserving tissue structure during the fixation process. It is known that RNA is fragmented by the fixation process. This method does not require decrosslinking of the samples. The whole transcriptome probes are 25 bp and able to hybridize to the fixed RNA inside the permeabilized nuclei/cells.

Our evaluation revealed consistent expression of cell marker genes and cell abundance, alongside strong correlation of pathway activities and similar characterizations of clinical condition between scFFPE-seq and fresh scRNA. Most importantly, we found that the expression of cell marker genes was broadly consistent across all cell types and tissues from both technologies. This indicates that scFFPE-seq can serve as a reliable alternative to fresh scRNA. However, it is important to exercise caution when studying specific cell populations, as some marker genes were hardly detected in FFPE-seq, such as CD8B for CD8 T cells, which aligns with previous studies involving FFPE and frozen samples [5,6]. At the gene signature levels, we observed considerable similarities in pathway activities across tissue types, such as TNFα and the JAK/STAT pathway, as well as clinical conditions when comparing HS and normal skin samples using scFFPE analysis, which mirror findings from fresh RNA analysis studies [22,23,24]. This provides strong evidence that scFFPE effectively captures the biological and clinical transcriptome features at the gene level. Together, these results suggest that scFFPE-seq could be a valuable tool for single-cell transcriptomics profiling, particularly when fresh samples are unavailable or difficult to obtain.

There are a few limitations that need to be acknowledged. First, the use of only one sample per tissue type and the limited number of cells recovered may have reduced the sensitivity of the results. With only one sample per tissue type, results may not account for biological variability. As a result, interpretations must be made cautiously, acknowledging that findings may not be generalizable until validated with larger, more diverse cohorts. Secondly, the absence of epithelium in the colon tissue restricted the scope of comprehensive comparison. Ideally, a direct comparison between FFPE samples and matched fresh scRNA samples would have been more informative, instead of relying on publicly available data. The discrepancy observed in keratinocytes from normal skin between our FFPE samples and fresh scRNA could stem from variations in study design within the public database [25]. The proportion of keratinocytes in our FFPE samples, through both scFFPE-seq and H&E estimation, was lower compared to the fresh scRNA data. This may be influenced by factors such as reduced cell recovery rates in normal skin. Additionally, since the IoU and F1 scores reported do not incorporate subjective errors, suggesting a potential over-representation of keratinocytes in the public data, which may not accurately reflect the true composition of the tissue. To clarify these observations, further investigation is necessary, specifically through direct comparison of scRNA results from FFPE and matched fresh tissue samples with large sample size. In addition, due to the increased degree of RNA degradation and fragmentation associated with FFPE processing, scFFPE-seq often yields lower gene detection counts and less comprehensive cell type coverage compared to other FFPE single-cell sequencing [9,10,11].

## 4. Materials and Methods

### 4.1. Sample Collection, scFFPE-Seq Library Preparation, and Sequencing

Formalin-fixed blocks of ileum, colon, and normal skin samples were purchased from Avaden Biosciences (Seattle, WA, USA) and Discovery Life Sciences (Huntsville, AL, USA) with consent from the participants. The Hidradenitis Suppurative (HS) sample was an excisional biopsy from the right axilla and has been previously described [24]. FFPE tissue sections were deparaffinized with three xylene washes (10 min each), followed by sequential washes in 100% ethanol (2×, 30 s each), 70% ethanol (30 s), and 50% ethanol (30 s) at room temperature. After brief washes in nuclease-free water and PBS on ice, PBS was removed and 2 mL Dissociation Enzyme Mix was added, ensuring scrolls were fully submerged. Tubes were sealed and processed on the gentleMACS Octo Dissociator (Miltenyi Biotec, Inc., Auburn, CA, USA) using program 37C_FFPE_1 (~48 min) with heating units. Following dissociation, tubes were centrifuged (300 rcf, 1 min), pellets resuspended, and the suspension filtered through a 30 μm Pre-Separation Filter into a chilled 15 mL tube. The gentleMACS tube (Miltenyi Biotec, Inc., Auburn, CA, USA) was rinsed with 2 mL chilled PBS to wash the filter and collect the filtrate. Samples were centrifuged (850 rcf, 5 min, 4 °C), supernatant removed, and pellets resuspended in 0.5 mL chilled Tissue Resuspension or Quenching Buffer, pipetted and mixed five times, and kept on ice.

Cells were isolated from FFPE scrolls using 10× Genomics protocol CG000632 for the gentleMACs Octo Dissociator (gentleMACs Program: 37C_FFPE_1). Four cell aliquots prepared for long-term storage were thawed at room temperature and then centrifuged at 850 RCF for 5 min at room temperature. The resulting pellets were resuspended in 500 µL of 0.5× PBS containing 0.02% BSA and 0.2 U/µL of RNase inhibitor. The pre-hybridization concentration of the FFPE dissociated suspensions were determined using Ethidium Homodimer-1 (Eth-1) on a Countess 3 FL (Thermo Fisher Scientific, Waltham, MA, USA). Between 400 k and 2 × 10^6^ cells are recommended for single-plex probe hybridization. One of the four samples fell below this guidance but was carried through for processing. Hybridization was carried out for 22 h, followed by 3 post-hybridization washes, and a post-hybridization cell count using Eth-1 on the countess 3 FL. The four cell suspensions were loaded into individual wells of a targeting cell recovery of 10,000. Remaining volumes of post-hybridized suspensions were preserved for long-term storage. Gel Bead in Emulsions (GEMs) were successfully generated and recovered from each sample. Four libraries were prepared with 14 sample indexing PCR cycles. Libraries were then quality checked, normalized, and pooled to a final loading concentration of 1.5 nM with a 1% PhiX spike-in. Sequencing was performed on the NovaSeq 6000 (Illumina, San Diego, CA, USA). Following 10× Genomics’ guidelines, the target sequencing depth was 10,000 read pairs per cell, utilizing a paired-end, dual indexing run.

### 4.2. H&E Staining and Image Analysis

Five-micron sections from all blocks were stained with Hematoxylin and Eosin (Leica Autostainer XL (ST5010), Wetzlar, Germany) and digitally imaged using the Panoramic 250 whole-slide scanner (3DHistech, Budapest, Hungary). H&E sections were qualitatively evaluated by a board-certified pathologist to determine tissue size and quality. Unstained scrolls were then sectioned at 50 µm (2 scrolls per colon and ileum samples and 6 scrolls per skin sample) and placed in 1.5 mL PCR Clean Eppendorf tubes (Eppendorf North America, Enfield, CT, USA) for the Chromium workflow (CG000477). RNA was extracted from FFPE scrolls using standard procedures. All samples had DV200 values > 30 (Qiagen’s AllPrep DNA/RNA FFPE Kit #80234, Qiagen, Germantown, MD, USA).

For the image analysis, whole-slide images (WSIs) of H&E-stained tissue sections were analyzed using Visiopharm software (version 2020.01.1.7332). A series of proprietary image analysis algorithms were applied to (1) detect the entire tissue area; (2) segment the epidermis in skin samples (normal and HS) and the epithelium in gastrointestinal (GI) samples (ileum and colon) to define regions of interest (ROIs); and (3) identify all nuclei within these ROIs and across the full tissue section. Cells identified within the epidermal and epithelial ROIs were interpreted as keratinocytes (skin) or epithelial cells (GI tract), respectively. For GI tissues, an additional proprietary deep learning algorithm was applied to detect neutrophils within the colon and ileum.

Epithelial segmentation was performed using a U-Net architecture trained on 225 WSIs at 5× magnification with manual epithelial annotations. Model performance was evaluated on a holdout set of 45 WSIs, achieving a mean Intersection over Union (IoU) of 0.910. Epidermal segmentation in skin samples was performed using a DeepLabv3 model trained on 51 WSIs with manually annotated epidermal boundaries and validated on a holdout set of 12 WSIs, yielding a mean IoU of 0.887.

Cell segmentation and classification were conducted using a separate U-Net convolutional neural network trained on 75 hand-annotated tiles (1024 × 1024 pixels at 20× magnification) derived from H&E-stained colon and skin tissues, with 20 tiles held out for validation. The model was trained to classify the following three categories: (1) background, (2) neutrophils, and (3) all other cells. Active learning was performed in 25-tile increments across three iterations, with expert pathologist input incorporated between rounds. The final model achieved a mean IoU of 0.885 for total cells (neutrophils + other cells) versus background, and an F1 score of 0.842 for neutrophil classification.

Quantitative outputs included the percentage of epithelial cells, keratinocytes, and neutrophils (in colon and ileum), each normalized to the total number of cells across the entire tissue section.

### 4.3. Selection of Public scRNA Data

To ensure the reliability and relevance of the reference data used in our study, we used publicly available data which provide comprehensive coverage of the respective tissue types, capturing both healthy and diseased states to ensure alignment with the biological diversity of our FFPE samples. The Crohn’s disease (CD) colon data utilized reference data from CD patient colon tissue, while the normal ileum data used reference data from the terminal ileum of healthy participants [26]. Skin reference database was integrated from multiple studies [22,23,24,25]. Reference data for HS (GSE155850, GSE154775, and GSE212721) and normal skin (GSE147482, GSE153760, EGAD00001004367, and E-MTAB-8142) were obtained from HS patients’ lesional tissue and skin tissue from healthy participants, respectively.

### 4.4. Bioinformatics Analysis

The data analysis flowchart is shown in Figure 1A. Sequencing reads were processed by 10× Genomics Cell Ranger 7.1.0 [3] with alignment of human GRCh38 reference genome. Cell calling was performed using the cellranger *multi* function as recommended by the *cellranger* tutorial. For each sample, we adapted an interquartile range (IQR) based filtering method [27] to remove low-quality cells. After normalization, doublets and multiplets were filtered out using DoubletFinder [28]. Statistical parameters (nExp and pK) were determined using the recommended settings of DoubletFinder, assuming doublet rate from Chromium Next GEM user guide (CG000204) to create artificial doublets for classification.

Cell types were annotated using a combination of manual and automated reference-based methods [29,30]. For automated annotation using SingleR version 2.0.0 [31], a public database with matched disease and tissue location served as the reference data (see Section 4.3 for method details). Additionally, we used manual annotation to identify cell types that were unique in FFPE but not present in the fresh samples as reference data, such as eosinophils, neutrophils, and adipocytes. Canonical cell markers were selected based on prior biological knowledge from literature [22,26], Seurat, and from CellMarker [32] (Supplementary Table S1). Lastly, gene markers from each cell type were also compared with a gene signature database for validation [16]. Seurat R package v 4.1.0 was employed for dimensional reduction, clustering [33,34], differential expression analysis, as well as gene signature module scores calculation [35] MSigDB [36].

## 5. Conclusions

In summary, our pilot study evaluated scFFPE-seq on challenging FFPE tissues and demonstrated its ability to recover unique cell types. The validation of scFFPE-seq data through H&E images and its comparability to fresh scRNA data highlights its effectiveness in capturing single-cell transcriptome. Further studies are needed to validate these findings. We recommend direct comparison of scRNA data from FFPE and matched fresh tissue. Using paired sample designs, where both FFPE and fresh tissues are collected from the same patient for single-cell sequencing, would help accurately assess scFFPE-seq’s reliability and capabilities.

## Figures and Tables

**Table 1:** Sequencing parameters for each sample.

	CD Colon	Normal Ileum	HS Skin	Normal Skin
Cells sequenced	16,130	4387	9091	1723
Cells passed QC	13,778	3720	7705	1406
Median reads per cell	7156	11,994	6208	25,061
Median UMI counts per cell	1753	2264	1474	1577
Median genes per cell	1150	1416	818	1044
Total genes detected	16,950	16,478	16,989	15,659
Confidently mapped reads in cells	94.90%	95.00%	90.83%	82.98%
Sequencing saturation	73.43%	79.68%	73.17%	93.11%

**Table 2:** Cell count and proportions identified by scFFPE-seq and H&E images.

	Epithelial Cells		Neutrophils		Keratinocyte	
	Colon	Ileum	Colon	Ileum	HS Skin	Normal Skin
H&E estimation	4738 (0.9%)	26,226 (17.2%)	1282 (0.2%)	357 (0.2%)	19,551 (9.3%)	1515 (16.1%)
scFFPE-seq	21 (0.2%)	609 (16.37%)	51 (0.2%)	106 (0.7%)	900 (10.8%)	395 (28.1%)

## Data Availability

The original contributions presented in this study are included in the article/Supplementary Materials. Further inquiries can be directed to the corresponding author.

## Supplementary Materials

The following supporting information can be downloaded at: https://www.mdpi.com/article/10.3390/ijms27031605/s1.
