# Supplementary material for: Probing the Feasibility of Single-Cell Fixed RNA Sequencing from FFPE Tissue
Source: Int J Mol Sci. 2026 Feb 6;27(3):1605. doi: 10.3390/ijms27031605 (PMC12898089; doi:10.3390/ijms27031605)
Supplement: Supplementary file 1 [file ijms-27-01605-s001.zip › Supplementary Figures.pdf]

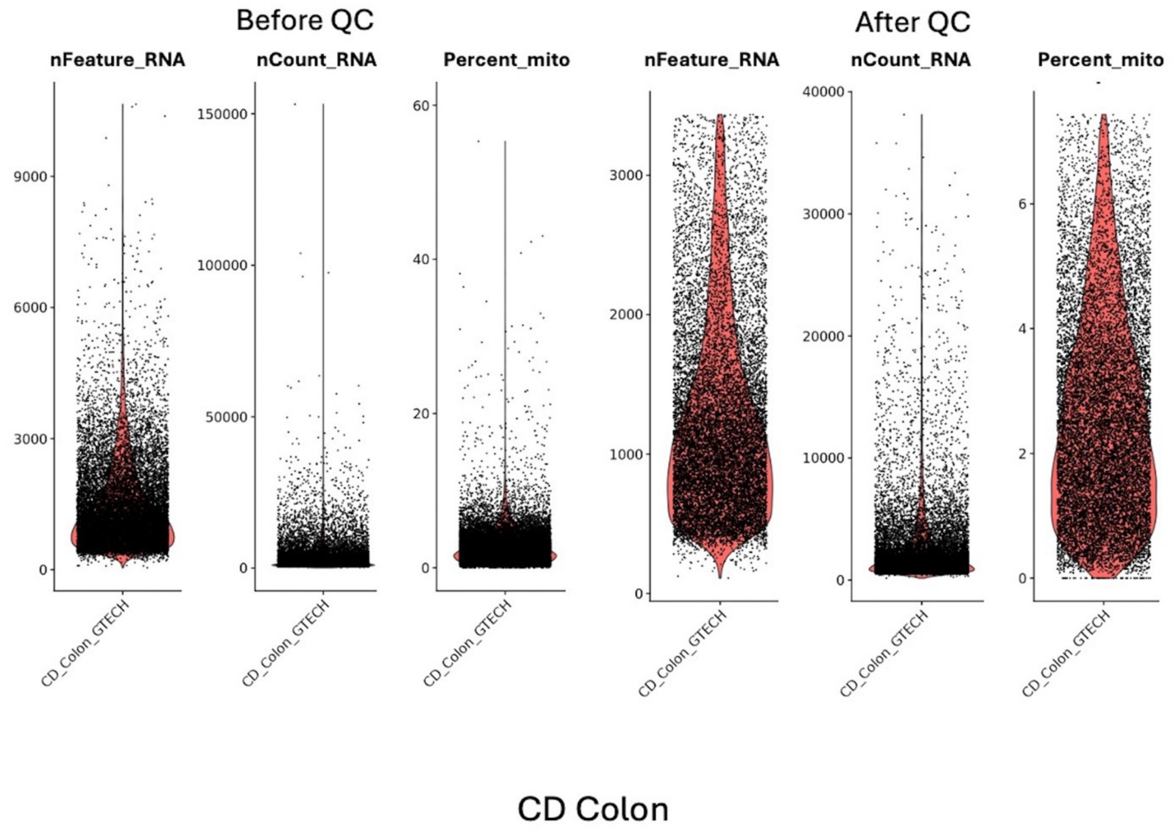

Figure S1: Violin plots of QC metrics before and after QC for CD Colon tissue

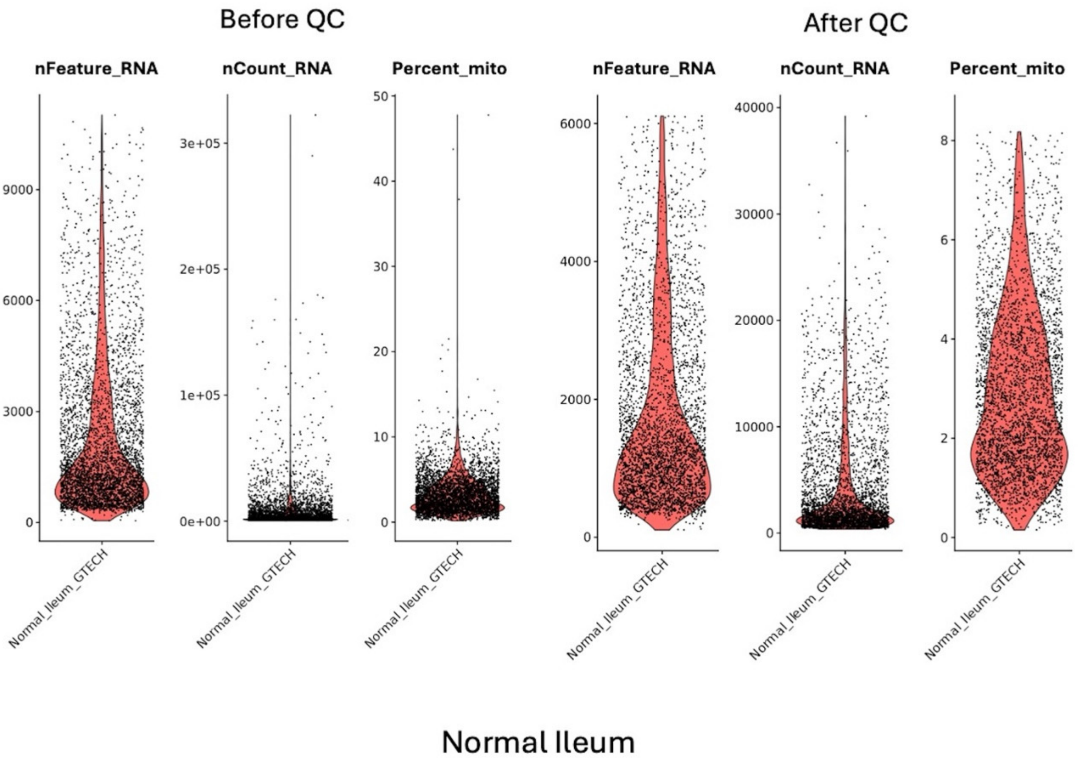

Figure S2: Violin plots of QC metrics before and after QC for Normal ileum tissue

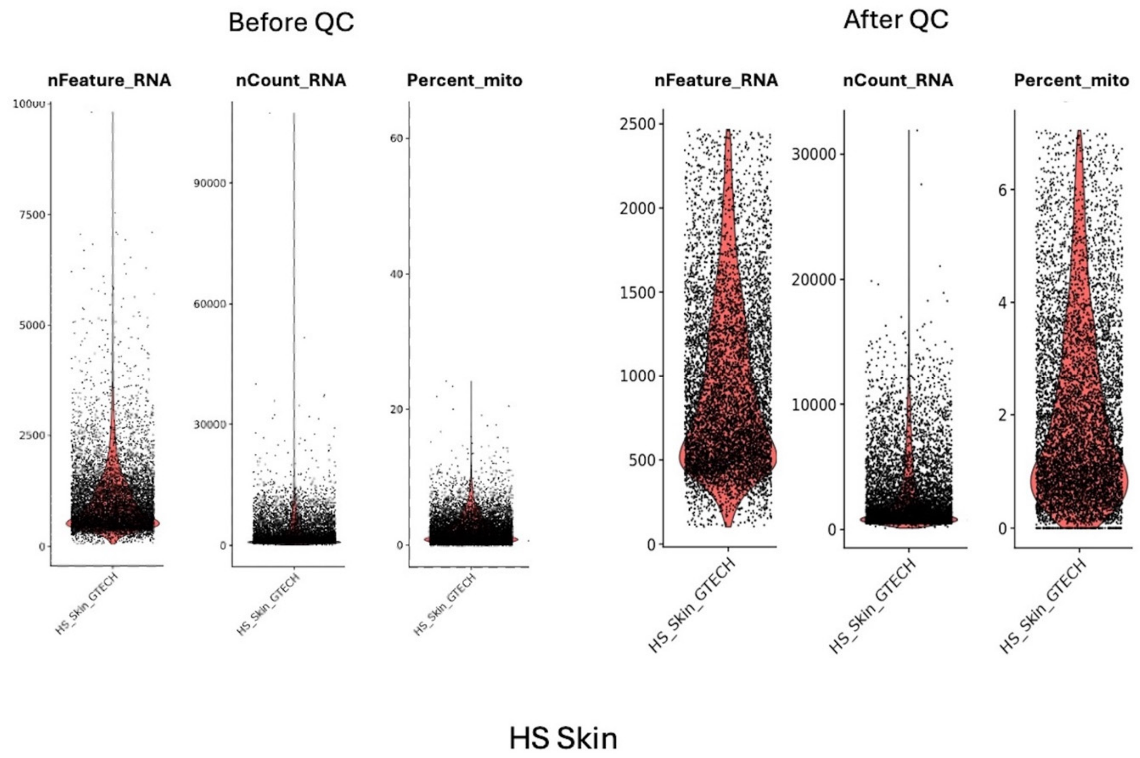

Figure S3: Violin plots of QC metrics before and after QC for HS skin tissue

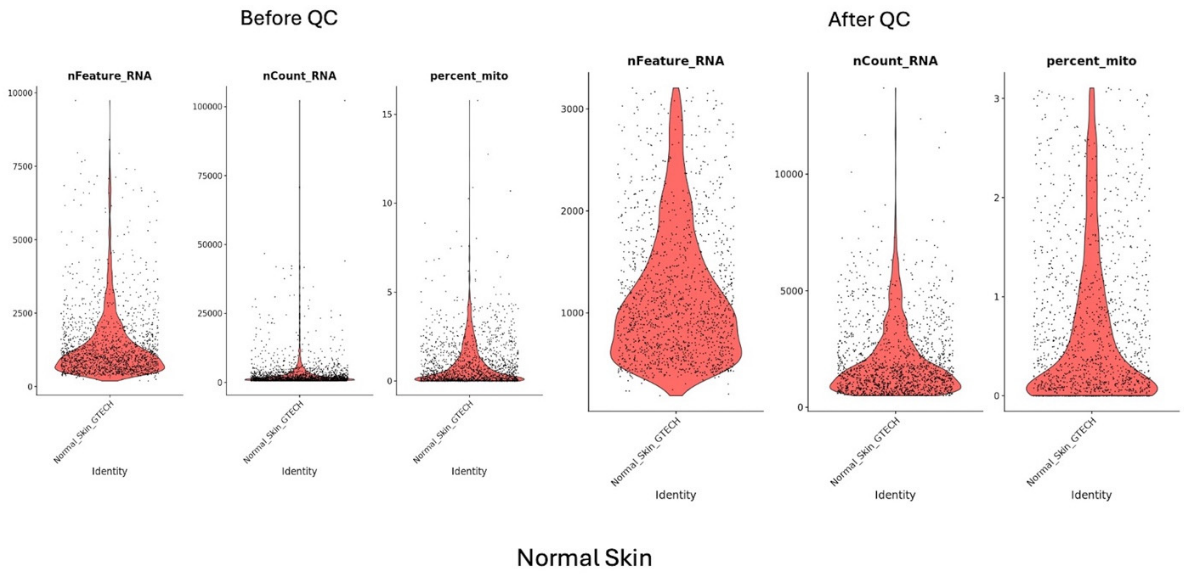

Figure S4: Violin plots of QC metrics before and after QC for normal skin tissue

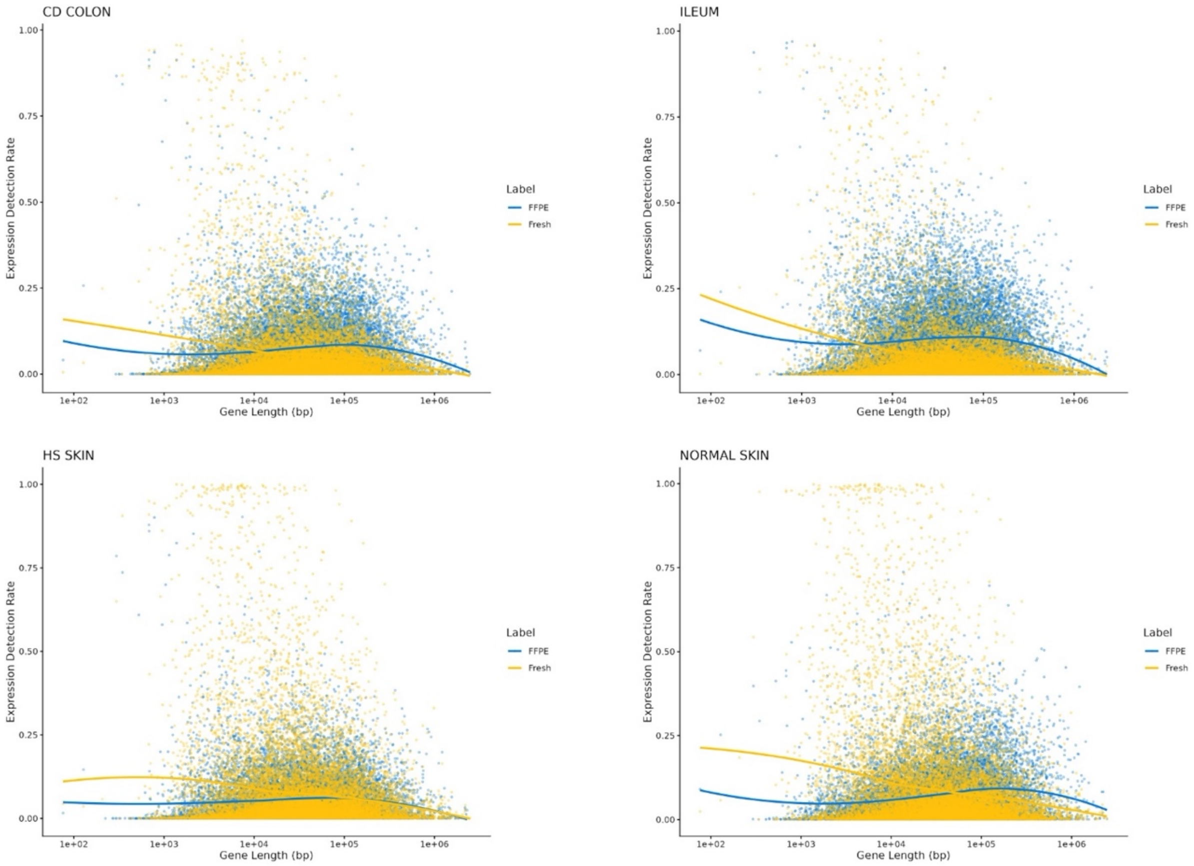

Figure S5: expression detection rate and gene length, colored by FFPE and Fresh across all 4 tissue types

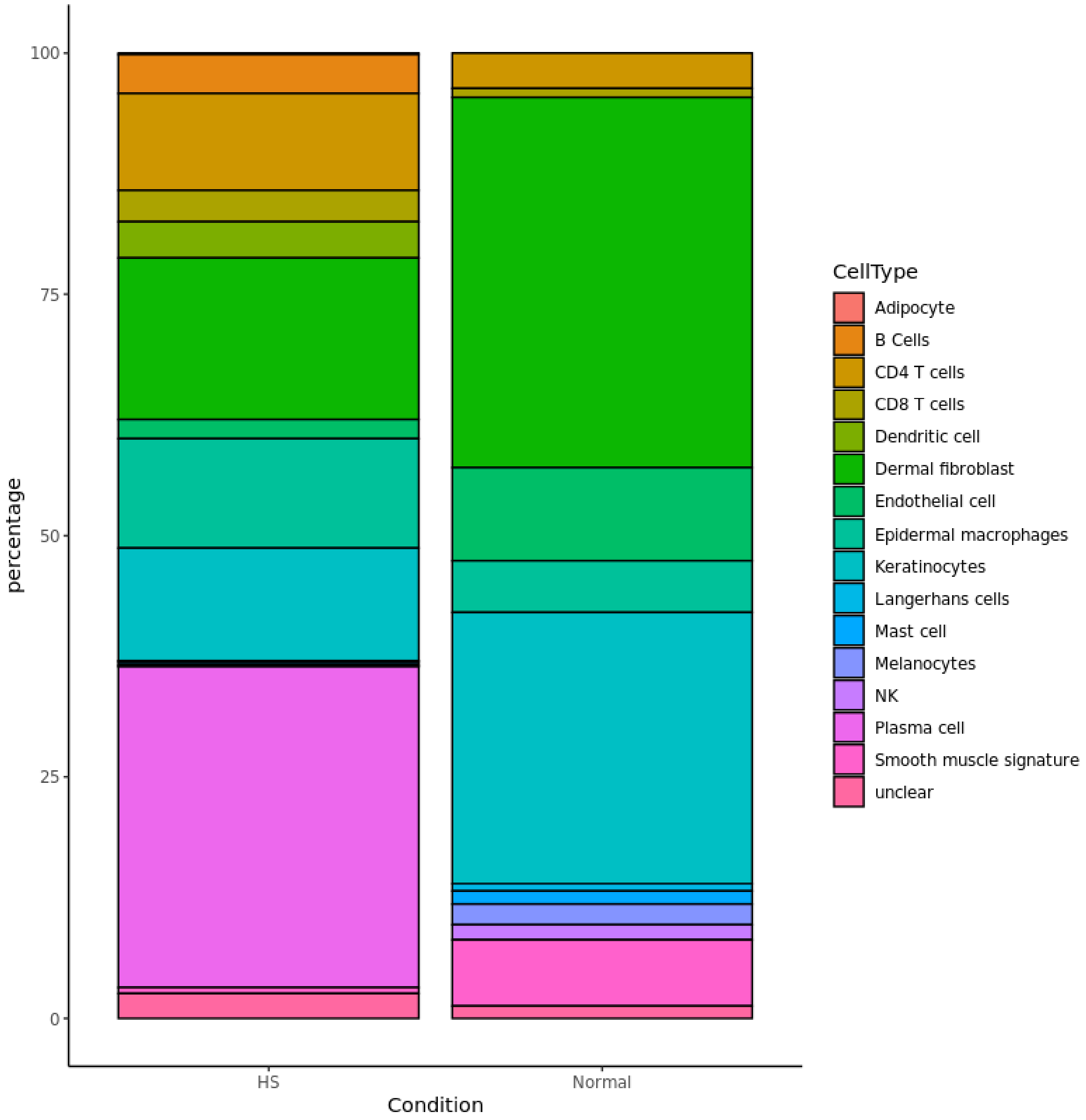

Figure S6: percentages of cell abundance for HS vs. normal from skin tissues, colored by cell types.

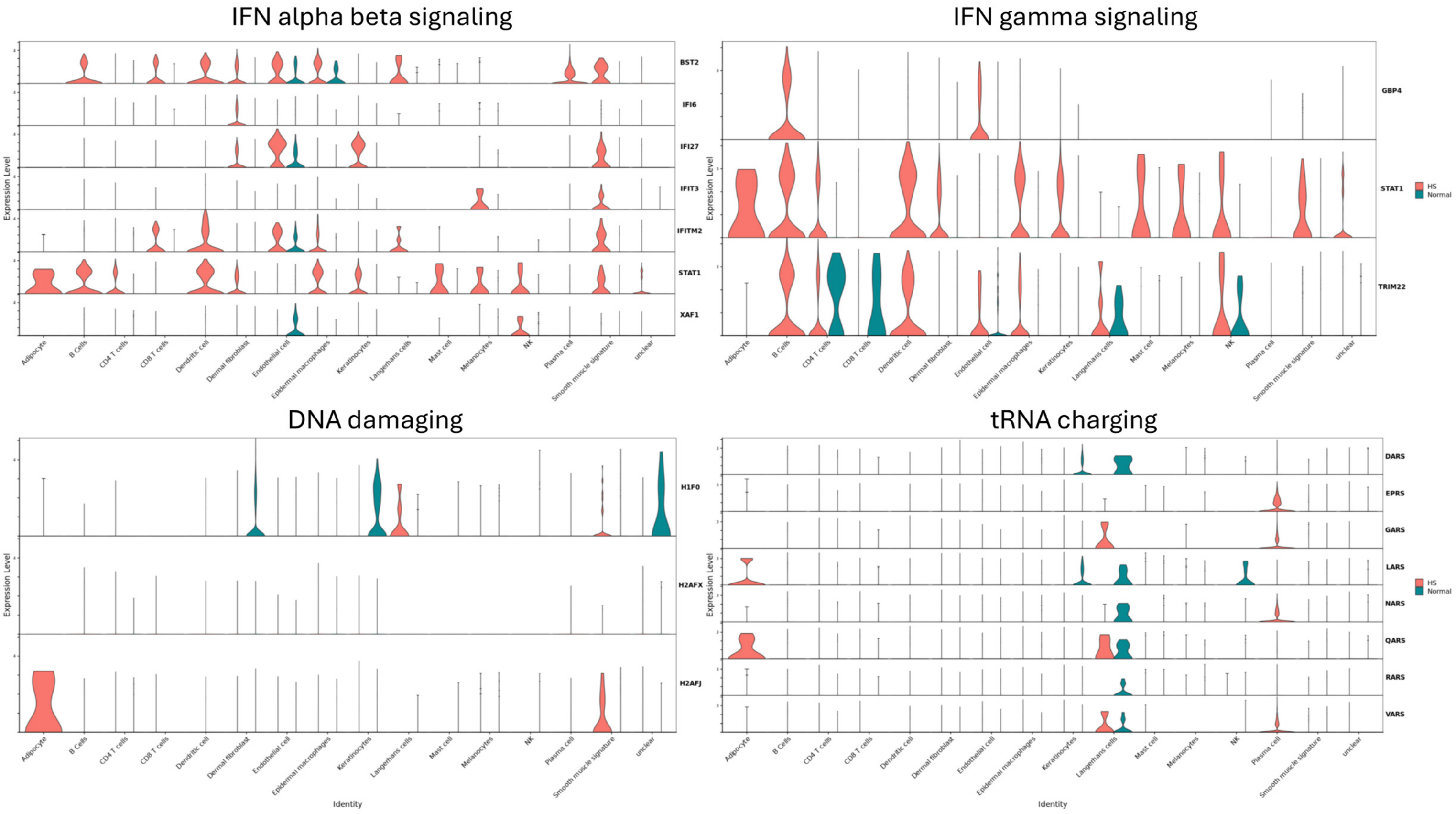

Figure S7. Violin plots of gene expression levels by cell types and condition on biological relevant selective pathways. X-axis indicates cell types, y-axis indicates gene expression levels. HS samples shown in red and normal shown in green.
